# Adaptive Catalytic Interfaces for Biomass-Derived Model Compounds: Dynamic Active Sites, Microenvironments, and Operando-Guided Design

**DOI:** 10.3390/molecules31193498

**Published:** 2026-10-01

**Authors:** Muhammad Saeed Akhtar

**Affiliations:** Department of Chemistry, Yeungnam University, Gyeongsan 38541, Republic of Korea; msakhtar@yu.ac.kr

**Keywords:** biomass valorization, adaptive catalysis, catalyst restructuring, operando spectroscopy, catalytic microenvironment, solvent effects, hydrogen spillover, interfacial catalysis, 5-hydroxymethylfurfural, lignin

## Abstract

Rational catalyst design for biomass valorization often relies on static descriptors, although water, hydrogen, electric potential, concentrated oxygenates, and reactive intermediates can reorganize catalytic interfaces during turnover. This review develops an adaptive-interface framework in which the relevant entity is the distribution of working states established under reaction conditions. Four coupled phenomena are integrated: reaction-induced restructuring; solvent-, confinement-, and wettability-controlled microenvironments; cooperative chemistry involving spillover, bifunctionality, and site proximity; and operando-to-model workflows. Studies of 5-hydroxymethylfurfural, furfural, lignin-derived oxygenates, and reductive catalytic fractionation show how phase, valence, hydration, hydrogen speciation, and interfacial proton transfer can redirect elementary pathways without changing nominal catalyst composition. Representative examples include nickel oxyhydroxide phase transitions, solvent-modulated active hydrogen, water-mediated hydrogen heterolysis, and metal–support coupling of hydrogen activation with selective C–O or C=O conversion. A closed-loop workflow combining operando spectroscopy, transient/isotopic kinetics, explicit-solvent computation, microkinetics, and data-driven exploration is proposed to map working states and guide synthesis. This perspective shifts rational design from optimizing a precatalyst toward engineering the interface present during reaction. Mechanistic evidence centers on molecule-defined substrates and lignin-derived models; lignocellulosic fractionation is included as process context. Here, operando denotes catalyst-state characterization during turnover with concurrent measurement of catalytic function, not operando analysis of intact biomass.

## 1. Introduction

Biomass valorization has matured from a search for catalysts that can merely transform oxygen-rich feedstocks into a problem of controlling networks of parallel and consecutive elementary steps. Lignin-first biorefining illustrates the point: catalyst selection cannot be separated from solvolysis, stabilization of reactive fragments, hydrogen availability, and carbohydrate preservation, because the product slate is generated by coupled chemistry rather than by one isolated bond-breaking event [1]. The same systems-level constraint appears in emerging metal-free, photo-, and electrocatalytic routes, where local charge, proton activity, adsorption geometry, and transport can determine whether a platform molecule is selectively upgraded or diverted to degradation products [2]. Consequently, a formulation described only by bulk composition is often mechanistically under-specified. Recent field syntheses have established the mechanistic importance of solvent effects [3], catalyst wettability [4], operando observables [5], and dynamic active sites [6]. Adaptive catalysis has been framed explicitly in terms of reversibility, rapidity, and robustness [7]. A CO_2_-responsive supported Ru catalyst demonstrated reversible selectivity switching for the biomass-derived substrate furfural acetone [8], while a related design uses the H_2_/CO_2_–formic-acid equilibrium to trigger adaptive hydrogenation [9]. Hydrogen spillover provides an additional route for coupling sites [10]. These developments motivate a design language in which the catalyst is treated as an interface exposed to a reactive chemical environment, not as an immutable solid.

The liquid phase is especially consequential because biomass molecules are multifunctional, strongly solvated, and frequently processed in water or mixed solvents. Confined water can differ from bulk water in density fluctuations, hydrogen-bond structure, dielectric response, and chemical potential; those changes can shift adsorption and activation barriers inside porous catalysts [11]. Atomistic treatments of water–solid interfaces likewise show that explicit solvent configurations may alter both the preferred adsorption state and the calculated transition state, making continuum descriptions insufficient for reactions that directly reorganize hydrogen bonds [12]. In practice, wettability controls whether organic substrates, water, and hydrogen-rich domains can simultaneously access active sites [4], while reactor architectures such as structured or three-dimensionally printed catalysts introduce an additional level of control over mass transfer, heat transfer, and spatially resolved functionality [13]. These observations are mechanistically linked: the local environment sets the chemical potential of reactants and reactive hydrogen, and the working catalyst adapts to that environment. Rational catalyst optimization therefore requires descriptors that couple material structure to interfacial composition rather than treating solvent and catalyst as independent variables.

Static catalyst taxonomies remain useful but can conceal the origin of selectivity. Hydrodeoxygenation reviews organize lignin-derived oxygenate chemistry around metals, phosphides, oxides, supports, hydrogen pressure, and acidity [14], whereas furfural hydrogenation is often compared through metal identity and product selectivity [15]. HMF hydrogenation/hydrogenolysis similarly emphasizes the balance among carbonyl hydrogenation, ring hydrogenation, and C–O cleavage pathways [16]. In electrochemical HMF upgrading, the same molecule can be driven toward oxidation or reduction products by potential, electrolyte, and electrode composition [17,18,19], and recent HMF electrooxidation surveys show a rapid expansion of catalyst classes and paired-electrolysis concepts [20]. Zeolitic biomass conversions add confinement and acid-site topology [21], while lignin depolymerization spans thermal, photonic, and electrochemical driving forces [22]. The common mechanistic problem is not lack of candidate catalysts; it is identifying which local state of which site carries a given elementary step under the reaction environment. Without that distinction, correlations between nominal composition and performance can be accurate empirically yet fail when feedstock composition, solvent activity, or scale changes.

The evidence base for this review was assembled as a targeted mechanistic literature screen rather than a formal systematic review. Peer-reviewed publications from 2019 through August 2026 were prioritized, with emphasis on original studies that directly linked catalytic performance to working-state structure, interfacial chemistry, solvent organization, hydrogen/proton transfer, defect chemistry, or operando characterization. Recent reviews of lignin-derived catalysts and structure–reactivity relationships were used to map established catalyst families and identify where primary mechanistic studies added information beyond compositional trends [23,24]. Bibliographic metadata and DOI information were cross-checked against publisher and indexing records. Greater emphasis was placed on studies that combined complementary mechanistic evidence, such as spectroscopy with kinetics, isotope labeling with product analysis, or atomistic modeling with experimental selectivity data. This evidence hierarchy is used throughout to distinguish observed working-state changes from proposed mechanistic assignments. Foundational studies published before 2019 were retained selectively when needed to interpret a mechanism or methodological principle. Mechanistic coverage centers on molecule-defined HMF, furfural, and lignin-derived oxygenates; whole-biomass and lignocellulosic-fractionation studies provide process context rather than the primary basis for operando assignments.

The aim of this review is to establish an adaptive catalytic-interface framework for rational biomass valorization in which the relevant design target is the working state generated under turnover rather than the synthesized precatalyst. Its objectives are to connect reaction-induced structural evolution with solvent and confinement effects; explain how spatially cooperative sites, hydrogen spillover, and interfacial proton transfer reshape elementary pathways; evaluate operando, transient, isotopic, and computational tools for resolving those states; and translate the combined evidence into testable design rules for HMF, furfural, lignin-derived oxygenates, and lignocellulosic fractionation. By organizing the literature around state evolution and interfacial function instead of catalyst labels, the review seeks to expose transferable descriptors that can guide synthesis, reactor operation, durability assessment, and scale-up. The intended outcome is a review architecture that allows readers to move from an observed catalytic trend to a specific interfacial hypothesis and then to an experiment capable of falsifying that hypothesis, rather than treating structure–activity correlation as an endpoint. Molecule-defined compounds provide the mechanistic core, while lignocellulosic fractionation is included to connect interfacial principles with process-level biomass conversion. The review’s contribution is this biomass-focused synthesis of state evolution, microenvironment, cooperative function, and operando-to-model evidence within a working-state framework.

## 2. From Static Active Sites to Adaptive Catalytic Interfaces

### 2.1. Defining the Working-State Ensemble

**Figure 1:**
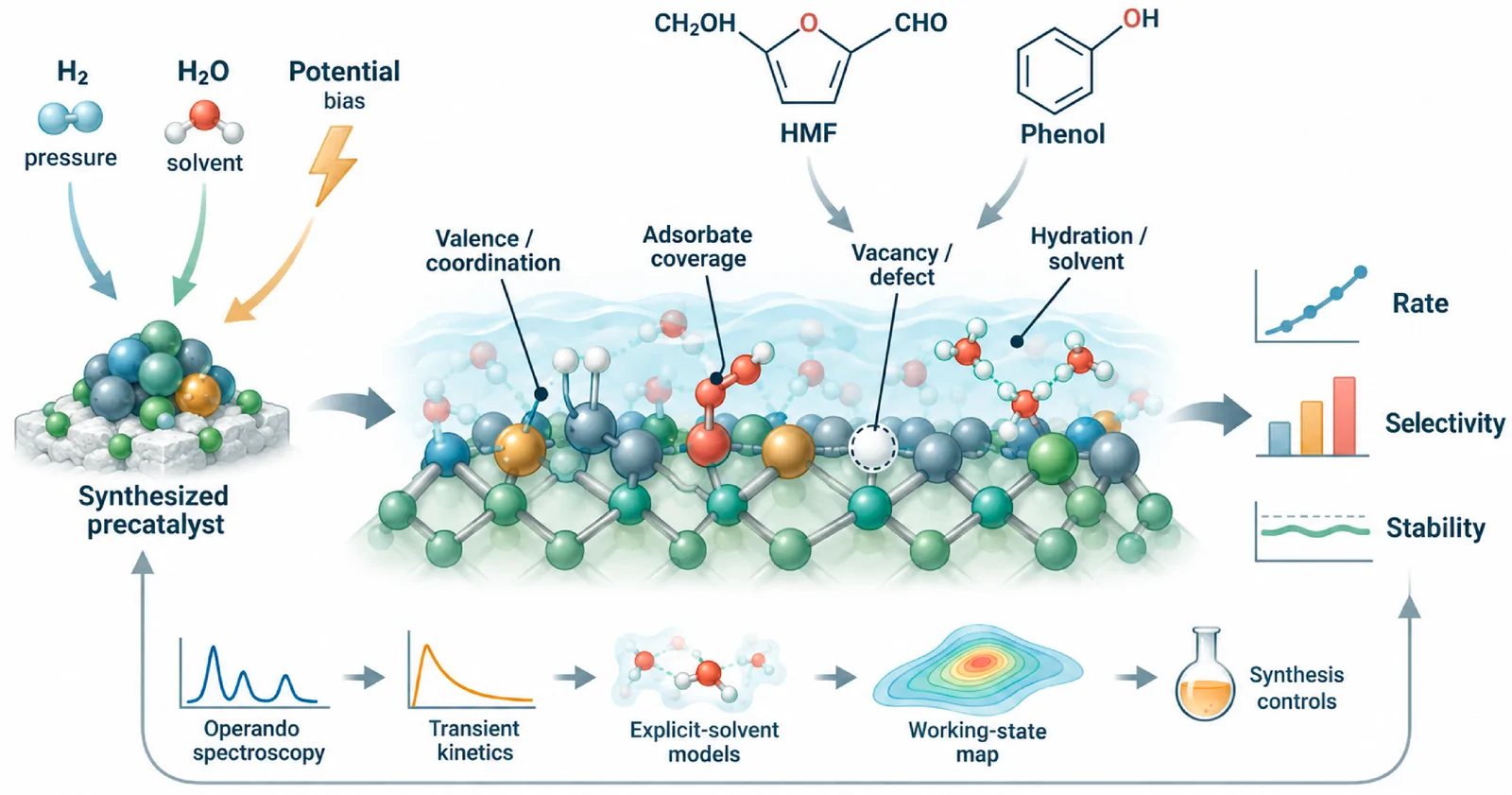
Adaptive catalytic-interface framework. The synthesized precatalyst is transformed by hydrogen, water, electric potential, biomass-derived adsorbates, and solvent into a distribution of working states. Operando measurements, kinetics, and modeling resolve which states control rate, selectivity, and stability; the resulting descriptors are then translated back into synthesis variables. HMF is shown as a furanic platform molecule, whereas phenol is a minimal aromatic oxygenate motif relevant to lignin-derived phenolics. Their inclusion makes the molecule-defined model-compound scope explicit; neither structure is intended to represent the full complexity of biomass.

An adaptive catalytic interface can be defined as an active-site ensemble whose composition, coordination, coverage, solvation, or spatial coupling changes measurably in response to the chemical potential imposed by the reacting medium. This definition is deliberately broader than conventional reconstruction because a catalyst can adapt without undergoing a bulk phase transformation. A nickel hydroxide surface may change oxidation state and adsorbate coverage with potential; a supported metal may acquire hydroxylated perimeter sites in water; a porous catalyst may retain a compositionally distinct solvent layer; and a bifunctional material may shift the dominant reaction center when hydrogen coverage changes. Dynamic-site concepts developed in electrocatalysis emphasize that the active state can be created only under bias [6], whereas operando biomass spectroscopy demonstrates that molecular intermediates and catalyst vibrations can be followed while current flows [5]. The practical consequence is that catalyst identity should be reported as a state distribution conditional on temperature, potential, pressure, solvent, concentration, and time. A single ex situ spectrum is then a boundary condition, not a complete mechanistic descriptor. Figure 1 summarizes this shift from precatalyst-centric to working-interface-centric design. In this review, “adaptive catalytic interface” denotes the broad working-state framework; “adaptive catalyst” is reserved for systems with demonstrated rapid, reversible, and robust responses to changing conditions, consistent with prior framing [7]. The framework therefore includes reaction-conditioned interfaces without implying that each reviewed catalyst was deliberately programmed to adapt.

The need for working-state descriptors is particularly clear for nickel oxyhydroxide electrocatalysts. Comparative studies of Ni, Co, and Fe oxyhydroxide anodes established that HMF oxidation activity and product formation depend strongly on the redox-active oxyhydroxide state rather than simply on the parent metal [25]. Subsequent mechanistic analysis showed that HMF oxidation on NiOOH can proceed through hydrogen-atom-transfer and hydride-transfer chemistry and that the composition of NiOOH alters the balance between electrochemical and chemical steps [26]. These observations imply two coupled clocks: the rate at which potential generates the oxidized nickel state and the rate at which the organic substrate chemically reduces or otherwise transforms it. If those clocks are comparable, the steady state is a dynamic mixture rather than a uniform phase. Catalyst comparisons made at fixed geometric current or nominal potential can therefore confound intrinsic organic oxidation kinetics with different fractions of the active nickel state. A rational descriptor must include the population and accessibility of that state during substrate turnover.

Cooperative materials make the state problem even more explicit because each component can carry a different elementary step. In a NiOOH-Cu(OH)_2_ system, alcohol- and aldehyde-group oxidation of HMF were assigned to complementary functions, enabling collaborative conversion toward FDCA [27]. Such behavior is not adequately represented by averaging metal ratios; the mechanistic variable is whether the two working states coexist at the interface, exchange intermediates rapidly enough, and remain accessible under the same potential and local pH. The same logic applies to thermocatalytic metal–acid pairs, metal–oxide perimeters, and encapsulated catalysts. The adaptive-interface concept therefore treats site proximity, coverage, and state synchronization as first-class descriptors. A material with excellent isolated functions can still underperform if an intermediate must desorb into bulk solution before reaching the second site, if one component blocks the other under realistic feed concentration, or if the local solvent stabilizes an off-cycle state. These failure modes are invisible when only composition and conversion are tabulated.

### 2.2. State Accessibility as a Synthesis Target

Working-state thinking also changes how stability should be interpreted. Activity loss can arise from irreversible sintering or leaching, but it can also reflect a reversible shift in phase, oxidation state, adsorbate population, or solvent occupancy caused by changing feed composition. Conversely, an apparently stable bulk structure can hide turnover-relevant restructuring confined to the first coordination shell. Operando phase-transition measurements on amorphous nickel oxyhydroxide during HMF oxidation directly demonstrate that the catalyst can move between NiOOH- and Ni(OH)_2_-like states on the reaction time scale [28]. Thin-film nickel studies likewise emphasize the value of controlling catalyst geometry so that observed current and chemical-state changes can be connected to a tractable surface inventory [29]. The design question is therefore not simply whether the catalyst changes, but whether the transformation is reversible, whether it creates the productive site, and whether the rate of state interconversion limits turnover. A durable adaptive catalyst can repeatedly traverse productive states; a deactivating catalyst accumulates inaccessible or nonproductive states.

For synthesis, the implication is to target state accessibility rather than a single nominal structure. Defects, strain, heteroatom coordination, support reducibility, and pore chemistry should be selected according to how they alter the free-energy landscape connecting precatalyst and working states. Cation-defective nickel hydroxides, for example, can facilitate conversion to active NiOOH while simultaneously altering HMF adsorption [30]. Strain engineering has been used to promote in situ NiOOH formation in Co-containing systems [31], and ligand-derived coordination environments can enrich NiOOH intermediates during HMF electrooxidation [32]. These strategies illustrate a general principle: a precursor is valuable when it enters, stabilizes, and exits the productive state at the correct rates. Designing only the lowest-energy ex situ phase can be counterproductive if that phase is kinetically trapped away from the catalytic cycle. A practical screening metric is therefore the hysteresis of state formation and removal during repeated potential, pressure, or solvent cycles. Small hysteresis together with recovery of the catalytic state and selectivity may indicate a readily reversible transition, whereas large hysteresis may reflect kinetic trapping, metastability, transport-related memory effects, or irreversible structural change.

## 3. Reaction-Induced Restructuring in Biomass Catalysis

### 3.1. Types and Timescales of Restructuring

**Figure 2:**
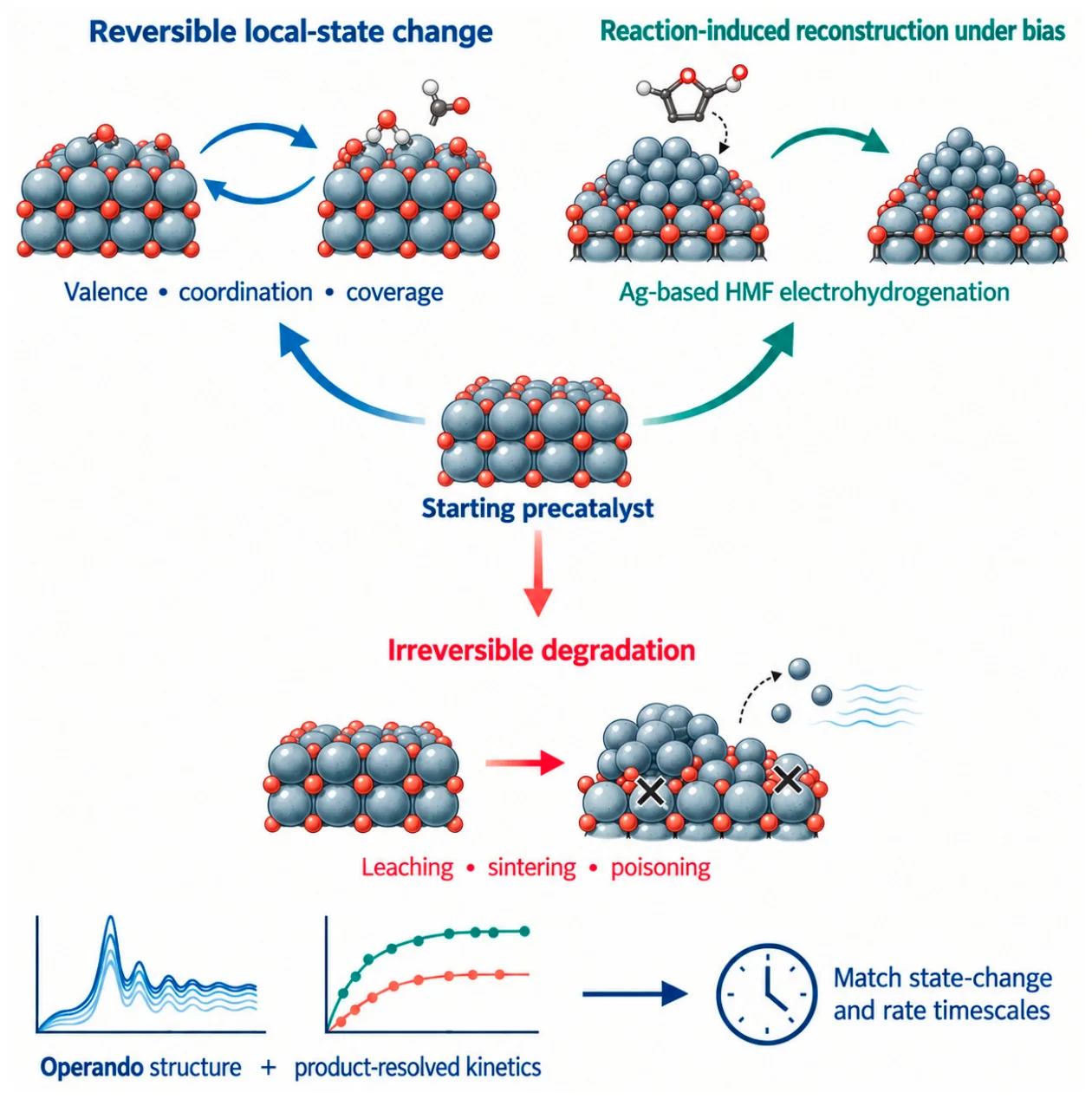
Reaction-induced restructuring pathways relevant to biomass catalysis. Working-state transformations can involve reversible local-state changes, reaction-induced reconstruction under bias, or irreversible degradation, depending on the pathway and reaction conditions. Mechanistic assignment requires linking state changes to kinetic response on comparable timescales.

Reaction-induced restructuring spans electronic, coordination, and morphological length scales. At the smallest scale, oxidation-state cycling changes adsorption strengths and the accessibility of proton-coupled electron transfer. At larger scales, hydration, vacancy generation, dissolution-redeposition, segregation, and phase transitions create new ensembles or destroy old ones. In nickel oxyhydroxides, even mechanistic assignments developed for the competing oxygen evolution reaction are relevant because the coordination and valence requirements of NiOOH determine which nickel atoms are oxidizing organic substrates [33]. The key is not to assume that a descriptor derived for water oxidation transfers unchanged to biomass oxidation; rather, substrate adsorption and chemical reduction of high-valent nickel can reshape the steady-state population. This feedback is central to adaptive catalysis. An organic molecule is not merely a reactant probing a fixed surface: it can be a reductant, ligand, proton donor/acceptor, and restructuring agent. Figure 2 groups the transformations that should be considered when a biomass catalyst is moved from synthesis conditions to realistic turnover. Figure 2 organizes these changes by scale: valence, coordination, and adsorbate coverage alter local reactivity, whereas dissolution–redeposition, phase change, and sintering change the available ensemble. A reversible arrow should be assigned only when the state returns after restoration of the perturbation and the kinetic response follows on a comparable timescale; operando NiOOH phase tracking during HMF oxidation illustrates why temporal coupling matters [28,29].

Electroreductive HMF chemistry provides a complementary example in which restructuring and cooperative interfaces determine hydrogenation. Ag/SnO_2_ was reported to broaden the potential window for HMF electrocatalytic hydrogenation through cooperation between the silver and oxide components [34]. A later study showed that silver can reconstruct in situ and that the reconstructed surface exhibits competent HMF hydrogenation activity [35]. These findings caution against assigning selectivity to the as-synthesized silver facet or particle morphology alone. Under cathodic bias, adsorbed hydrogen, hydroxide, organic intermediates, and local cation fields can drive changes in roughness, coordination, or oxide coverage; the state that binds HMF may therefore be generated only after polarization. The mechanistic design lever is the pathway into the active surface state and its persistence across the intended potential window. Ex situ microscopy before and after electrolysis can establish net morphology changes, but it cannot resolve transient states that form and disappear within a catalytic cycle. Coupling operando structural probes with product-resolved electrokinetics is required to separate productive reconstruction from damage. Figure 2 places this HMF example on the reaction-induced reconstruction pathway: cathodic polarization can alter the Ag surface, while product-resolved electrokinetics can determine whether the resulting state contributes to productive hydrogenation or degradation [34,35].

Rapid reconstruction can also be deliberately exploited rather than suppressed. Amorphous surfaces contain a broad distribution of coordination environments and may transform more readily than crystalline analogues. In HMF-assisted hydrogen-production systems, rapid surface reconstruction has been linked to enhanced organic oxidation coupled with hydrogen evolution [36]. Similarly, interfacial proton transfer can be accelerated by installing Brønsted-basic functionality near the catalytic surface; such functionality alters the kinetic accessibility of proton-coupled steps without requiring a new bulk catalyst phase [37]. These examples extend the definition of restructuring beyond crystallographic phase change. A surface that dynamically changes protonation, hydroxylation, or ligand coordination can produce a different transition-state ensemble even if diffraction shows no new phase. Mechanistically, the relevant evidence is a correlated change between local state and rate/selectivity under the same perturbation. Temperature, potential, isotope substitution, or controlled ligand/base addition can provide that perturbation and help distinguish causation from a merely coincident structural signal.

Selective partial oxidation illustrates why the identity of the working oxidant must be resolved. NiAl layered double-hydroxide nanosheets have been used to direct HMF toward 2,5-diformylfuran rather than complete oxidation, demonstrating that catalyst structure can alter which functional group is oxidized at a given potential [38]. In related polyol electrooxidation, operando tracking of surface *OH on heteroatom-tailored Co_3_O_4_ connected hydroxyl coverage with high-current-density ethylene glycol oxidation [39]. The transferable lesson is that surface oxygen species should be treated as dynamic reagents with coverage-dependent chemistry, not as passive spectators. High coverage can increase the probability of oxygen-transfer or hydrogen-abstraction events, yet it can also block organic adsorption or accelerate C-C cleavage. Therefore, an optimal catalyst may require a narrow window of oxidized-state population rather than maximal oxidation. This window should be mapped against substrate concentration and current density because the organic reactant itself consumes or displaces surface oxygen species.

### 3.2. Distinguishing Productive Dynamics from Degradation

Dynamic-state analysis is equally important on the reductive side of biomass electrochemistry. HMF has been used as a model system to show that electrochemical hydrogenation, hydrogenolysis, and dehydrogenation can be selected by controlling electrode material and potential [40]. Furfural electrohydrogenation studies using biomass-derived electrolytes further demonstrate that electrolyte composition inherited from upstream processing can change conversion behavior relative to purified model solutions [41]. These observations expose a common scale-up risk: a catalyst optimized in a simple supporting electrolyte may enter a different working state in an authentic biomass liquor containing salts, acids, phenolics, and metal ions. Competitive adsorption can change hydrogen coverage; trace ions can deposit or coordinate; and pH gradients can alter surface speciation. A robust adaptive catalyst should therefore be characterized under progressively more realistic feeds. The criterion for transferability is not preservation of the pristine structure, but preservation of the productive working-state distribution and its selectivity under impurity and concentration perturbations.

Thermocatalytic systems undergo analogous state changes, driven by pressure, solvent chemical potential, temperature, and adsorbate coverage rather than electrode potential. Adaptive Ru/support studies show that changing the H_2_/CO_2_ feed can reversibly switch hydrogenation selectivity [8,9]. The biomass-derived substrate furfural acetone was used in one demonstration [8]. This evidence establishes feed responsiveness; hydroxylation, vacancy generation or filling, and acid-site hydration remain catalyst-specific hypotheses until linked to structural and kinetic measurements. Because state conditioning can evolve during a batch run, induction periods must be distinguished from steady-state kinetics. If selectivity improves after an initial period, the catalyst may be conditioning into a productive state; if selectivity drifts, the interface may be evolving continuously. Time-resolved sampling combined with before/after characterization is a minimum requirement, while operando X-ray or vibrational probes can test whether changes in coordination, oxidation state, or adsorbate populations coincide with the kinetic drift.

A useful classification separates restructuring into reversible catalytic breathing, quasi-reversible conditioning, and irreversible degradation. Reversible breathing includes redox cycling, protonation, hydration, and adsorbate-dependent coordination that returns when the perturbation is removed. Quasi-reversible conditioning includes transformations that persist during a run but can be reset by regeneration, such as controlled reduction, vacancy formation, or ligand loss. Irreversible degradation encompasses sintering, deep leaching, pore collapse, and phase segregation that reduce the accessible productive ensemble. This classification should be reported alongside conventional stability metrics because a constant conversion can mask compensating changes in site number and intrinsic activity. Conversely, a changing spectroscopic signal does not necessarily indicate deactivation if the transformation is part of the catalytic cycle. The decisive experiment is perturbation-response: intentionally change potential, hydrogen pressure, water activity, or substrate concentration and determine whether the structural signal and rate respond reproducibly and reversibly. Such state-response maps are more informative for design than a single post-reaction micrograph. This three-way taxonomy is an organizing framework proposed in this review rather than a universal classification; its boundaries should be assigned using evidence for perturbation, recovery, and the corresponding kinetic response [6,7,8,9].

## 4. Solvent, Confinement, and Interfacial Microenvironments

### 4.1. Solvation and Hydrogen-Bond Control

**Figure 3:**
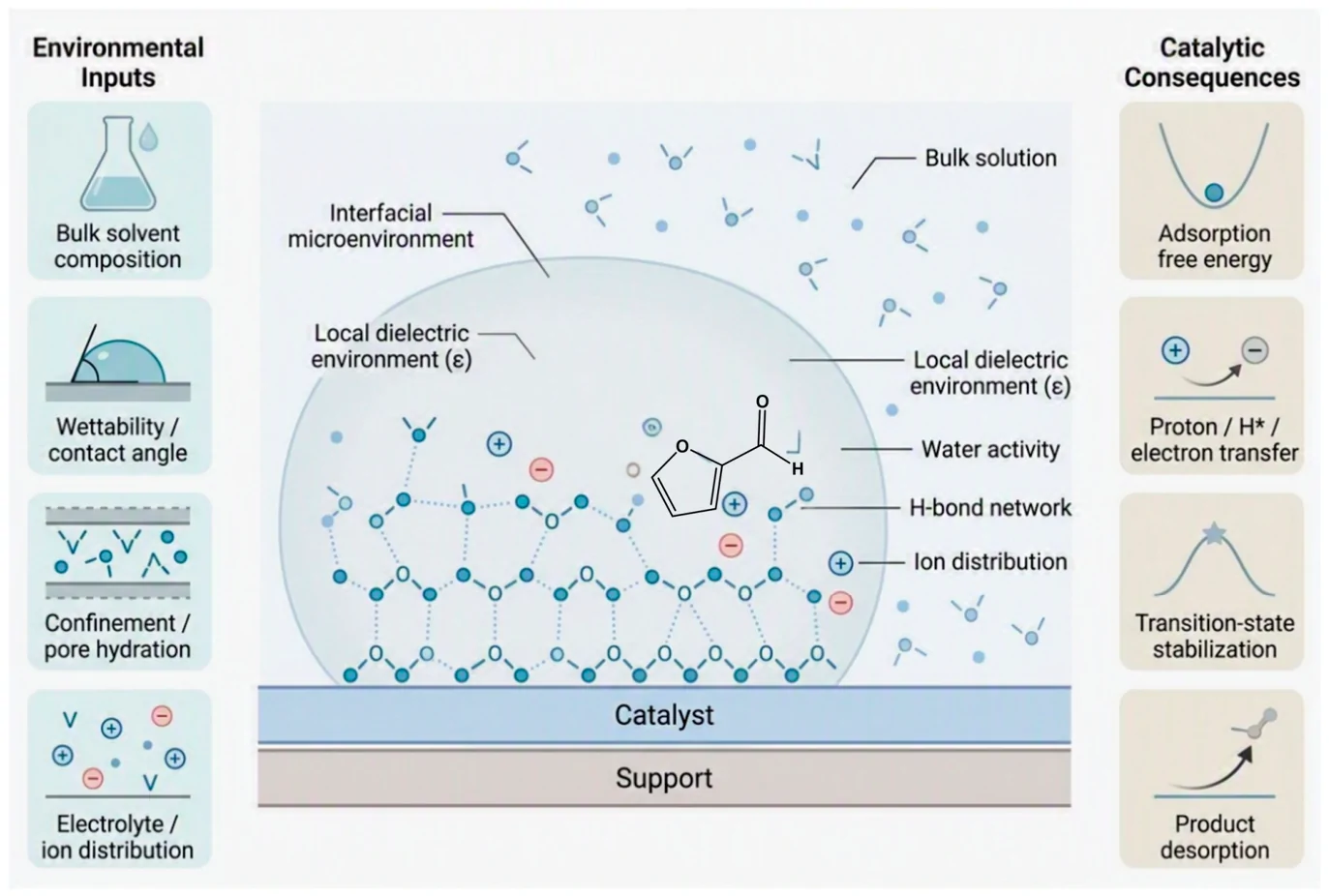
Microenvironment engineering at liquid–solid catalytic interfaces. Bulk solvent, wettability, confinement, and ions establish a local hydrogen-bond and electrostatic environment that changes adsorption, proton/hydrogen/electron transfer, transition-state stabilization, and product desorption. * denotes the transition state.

In liquid-phase biomass conversion, solvent molecules participate in the reaction coordinate by reorganizing adsorption, charge separation, hydrogen bonding, and proton mobility. Furfural hydrogenation provides direct evidence: solvent-dependent experiments and theory have identified multiple active hydrogen species whose relative populations change with the medium, producing distinct hydrogenation behavior [42]. Explicit simulations of furfural at metal–water interfaces further show that solvation changes adsorption configurations and reaction energetics compared with vacuum or implicit-solvent pictures [43]. The mechanistic unit is therefore not simply metal plus substrate; it is metal–substrate–solvent, with interfacial water able to compete for sites and stabilize polar transition states. Figure 3 represents this local environment as an adjustable free-energy field created by solvent composition, wettability, confinement, and ions. A useful experimental consequence follows: solvent effects should be interpreted with measurable interfacial descriptors, such as water activity, contact angle, adsorption enthalpy, or spectroscopic hydration signatures, rather than only bulk dielectric constant or polarity scales.

Hydrogen-bond engineering can be used deliberately to redirect selectivity. Organic modifiers on metal surfaces have been shown to promote furfuryl-alcohol ring hydrogenation through surface hydrogen-bonding interactions [44]. This result is significant because the modifier is not merely blocking sites; it changes how the substrate approaches the surface and stabilizes particular adsorbed geometries. Similar logic applies to support hydroxyls, adsorbed water, and tethered functional groups. More generally, appropriately positioned hydrogen-bond donors or acceptors can alter substrate adsorption geometry and transition-state stabilization, thereby influencing hydrogenation selectivity without necessarily changing the intrinsic hydrogen-dissociation capability of the metal. The design variable is thus the geometry and lifetime of the interfacial hydrogen-bond network. Excessive functionalization can, however, suppress rate by excluding reactants or hydrogen. Mechanistic optimization requires separating changes in apparent activation energy from changes in accessible site density, ideally through kinetic orders, adsorption measurements, and spectroscopy under the same solvent composition. This geometry-mediated route is illustrated in Figure 3 [44].

HMF hydrogenation over copper-based catalysts demonstrates that the support-metal interface and solvent jointly select pathways. Interfacial structure was shown to determine reaction route and selectivity in Cu-based HMF hydrogenation [45], indicating that a nominal Cu loading is insufficient to specify the catalytic ensemble. Furfural hydrogenation can be made highly selective by modularizing hydrogen dissociation and substrate activation on different functions [46]. PtNi alloy/SBA-15 systems likewise illustrate how alloy composition and aqueous operation can support selective carbonyl hydrogenation under mild conditions [47]. Across these examples, the microenvironment controls whether the oxygenated group is activated through Lewis-acid coordination, hydrogen bonding, or direct metal adsorption, while the metal controls the supply and reactivity of hydrogen. The optimum interface therefore matches substrate activation strength to hydrogen delivery. Too strong carbonyl binding can trap intermediates; too high hydrogen activity can promote ring saturation or hydrogenolysis; and insufficient interfacial polarity can lower aqueous accessibility. These are coupled, not independent, optimization variables.

### 4.2. Transfer Hydrogenation and Confined Environments

Transfer hydrogenation highlights solvent participation even more directly because the hydrogen donor is part of the liquid reaction network. Zr-based metal–organic frameworks can catalyze furfural transfer hydrogenation under mild conditions, with node coordination and modification influencing activity [48]. Hf-containing TUD-1 catalysts couple transfer hydrogenation with acid reactions of furfural and HMF, so acid–base and hydrogen-transfer functions must be balanced to avoid condensation or degradation [49]. Zirconium-carbon coordination catalysts have achieved efficient HMF-to-BHMF transfer hydrogenation [50], while Cu-Fe oxide systems can reduce HMF without molecular hydrogen [51]. These materials demonstrate that a catalyst’s effective hydrogen chemical potential can be generated locally from alcohols or other donors. The mechanistic descriptor should therefore include donor activation, hydride/proton transfer distance, and solvent competition for Lewis-acid sites. Comparing catalysts at the same nominal temperature while changing donor identity can reveal whether turnover is limited by donor dehydrogenation or by carbonyl reduction. Because donor oxidation products may themselves coordinate or change acidity, complete carbon balances and donor-product analysis are required. Rate comparisons should also distinguish true transfer hydrogenation from pathways in which donor decomposition first generates molecular hydrogen or another mobile reducing equivalent.

Confinement changes microenvironment by restricting both substrate orientation and solvent organization. Copper atom pairs embedded within a porous organic polymer cavity have been used to control HMF hydrogenation, linking a defined multinuclear motif to the cavity environment [52]. Ordered mesoporous carbon-supported platinum also shows that pore architecture, metal location, and solvent choice affect furfural hydrogenation and recyclability [53]. Membrane-reactor hydrogenation of furfural adds a reactor-scale form of confinement by separating hydrogen delivery from the bulk liquid phase [54]. In all three cases, the local concentration of hydrogen and oxygenate near the active site can differ from the bulk concentration. This means that external kinetic orders are effective parameters combining transport and chemistry. A mechanistic design strategy should quantify diffusion or permeation where possible, then use pore size, tortuosity, surface functionality, and hydrogen-delivery mode to establish a controlled interfacial composition rather than assuming the bulk liquid directly contacts an unconstrained surface.

Zeolitic and encapsulated systems provide a particularly useful test of confinement-spillover coupling. Platinum clusters encapsulated in amorphized HA zeolite have been reported to promote selective furfural hydrogenation through hydrogenation spillover [55]. Here the spatial relation between hydrogen-generating platinum and acid or framework environments becomes part of the catalytic pathway. Transfer-hydrogenation over Cu/ZnO/Al_2_O_3_ using methanediol as hydrogen donor [56], nickel-organoclay catalysts for deeper furfural hydrogenation [57], and Cu-Al_2_O_3_-ZnO catalysts for furfuryl-alcohol production [58] further show that support chemistry and hydrogen source jointly determine product depth. The practical lesson is that pore confinement should not be optimized only for adsorption capacity. It should be optimized for the desired sequence: arrival of the substrate, activation at the appropriate site, delivery of hydrogen or hydride, and rapid escape of the target product before secondary reactions occur. Pore-mouth chemistry is especially important because it can impose a second selectivity filter after the intracrystalline reaction. Measuring product diffusion or conducting size-selective probe reactions can reveal whether an apparent catalytic preference originates from transition-state stabilization inside the pore or from selective retention and secondary conversion of one product class.

### 4.3. Process-Level Solvent Effects

Solvent engineering must ultimately be evaluated at process-relevant concentration and impurity levels. Reviews focused specifically on furfural liquid-phase hydrogenation show that solvent effects can reverse activity and selectivity trends across catalyst families [59], while glucose-to-HMF studies demonstrate that Lewis-acid identity and organic solvent environment jointly determine HMF yield [60]. At high glucose loading, catalyst and solvent also influence HMF stability after it is formed [61]. Trace impurities can strongly perturb HMF hydroconversion [62], and molecular-dynamics-plus-experiment studies of xylose conversion show how solvent composition changes reactant organization even without a solid catalyst [63]. Integrated biomass-to-bioproduct process analysis further makes solvent selection a cross-unit-operation problem rather than a single-reactor choice [64]. Thus, a solvent that maximizes intrinsic rate may be inferior if it destabilizes product, poisons downstream biocatalysis, complicates separations, or changes catalyst working state with feed recycle. Microenvironment design should therefore be coupled to solvent recovery, impurity tolerance, and feed concentration from the outset.

## 5. Cooperative Interfaces: Spillover, Bifunctionality, and Proximity

### 5.1. Spillover as a Rate-Coupling Problem

**Figure 4:**
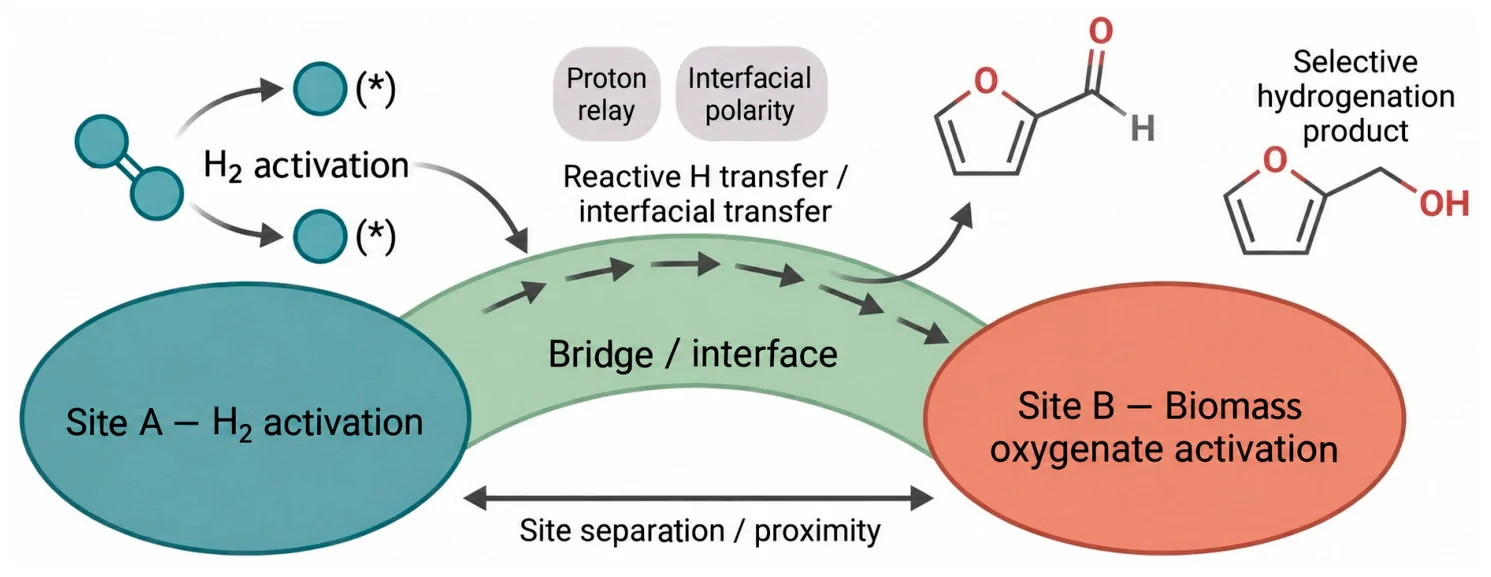
Cooperative catalytic interfaces. Site A generates activated hydrogen or another reactive equivalent, an interfacial bridge transfers it, and site B activates the biomass-derived substrate. Selectivity depends on matched rates, site proximity, interfacial polarity, and resistance to competitive adsorption. * denotes a surface-bound activated hydrogen species (H*) generated by H_2_ dissociation at Site A.

Spillover provides a mechanistic bridge between site chemistry and spatial catalyst architecture. In its classical hydrogen form, H_2_ dissociates on a metal and hydrogen species migrate to a support or neighboring site, extending reactive hydrogen beyond the geometric footprint of the metal [10]. For biomass hydrogenation, the important question is not whether spillover is possible in principle but whether its rate and travel distance are sufficient to compete with direct hydrogenation, recombination, solvent quenching, and product desorption under liquid-phase conditions. Organic molecular decoration has been shown to improve hydrogen-spillover efficiency and enhance catalytic hydrogenation performance [65], demonstrating that the bridge between source and sink sites can itself be engineered. Figure 4 frames this as a coupled-rate problem: site A generates an activated species, the interface transports it, and site B activates the substrate. Overall selectivity depends on matching these steps and preventing either site from becoming kinetically isolated.

Water can act as a functional component of cooperative interfaces rather than merely as solvent. Pd-S interfaces have been reported to catalyze hydrodehydroxylation of biomass-related chemicals through water-mediated hydrogen heterolysis [66]. In such a mechanism, the local arrangement of metal, sulfur-containing site, and water creates a pathway for heterolytic H_2_ activation that would not be described by metallic hydrogen coverage alone. This is a strong example of adaptive interfacial chemistry because hydration state changes the identity of the reactive hydrogen species. The design implication is to characterize acid–base functionality at the perimeter under water, including whether proton and hydride equivalents remain spatially coupled to the adsorbed oxygenate. Changing water activity or isotopic composition can test the role of interfacial proton transfer; changing the density of perimeter sites can test whether rate scales with interface length rather than metal surface area. Such experiments convert a qualitative ‘water effect’ into a mechanistic descriptor suitable for synthesis. This Pd–S example illustrates the interfacial H_2_-activation and reactive-hydrogen-transfer pathway summarized in Figure 4 [66].

### 5.2. Cooperative Chemistry in Lignin Conversion

Reductive catalytic fractionation illustrates cooperative chemistry at the scale of complex lignocellulose. Extremely low-loaded Pd catalysts can efficiently fractionate biomass when highly dispersed Pd species are stabilized on nitrogen-functionalized carbon, with product distributions linked to hydrogenolysis and double-bond saturation pathways [67]. Mechanistic work on lignin hydrogenolysis across plant clades emphasizes that the native polymer and associated phenolate structures define which linkages and intermediates reach the catalyst [68]. Independent reaction-mechanism studies of reductive catalytic fractionation show that solvolytic release and catalytic stabilization are temporally coupled [69]. The active interface is therefore not only metal–support; it includes the evolving lignin fragment and solvent. If fragment release outruns hydrogenative stabilization, condensation can lower monomer yield. If hydrogenation is too aggressive, valuable aromatic functionality may be lost. Catalyst design should consequently match hydrogenation capacity to the rate of lignin solvolysis under the chosen solvent and temperature rather than maximize hydrogenation activity in isolation.

Bifunctional metal–acid catalysts for lignin hydrodeoxygenation show a similar need for matched site functions. Ru/HZSM-5 couples metal hydrogenation with acidic chemistry to convert lignin and model compounds toward hydrocarbon fuels [70]. Ni-based catalysts can achieve efficient hydrodeoxygenation of lignin derivatives through a different balance of metallic and support functions [71], while hydrous ruthenium-oxide systems demonstrate that surface water molecules and support acidity can influence phenolic hydrodeoxygenation [72]. Hollow Ni-Fe architectures introduce additional metal–metal cooperation and transport control [73]. These systems should be compared through elementary-step responsibilities: which site activates H_2_, which adsorbs the phenolic oxygen, which cleaves C–O, and which promotes undesired ring hydrogenation or cracking. A composition may be called ‘bifunctional’ even when the two sites are too far apart to exchange intermediates efficiently. Spatial characterization and selective site poisoning are therefore essential for proving genuine cooperation. A rigorous proximity test can be created by physically mixing separately optimized metal and acid particles and comparing them with an integrated material at matched site inventories. A true interfacial advantage should persist after correcting for transport and should weaken systematically as the average distance between complementary sites increases.

Vapor-phase and condensed-phase lignin upgrading also reveal how support acidity and redox character reshape product distribution. MoO_3_/γ-Al_2_O_3_ catalyst properties were connected to product distributions during hydrodeoxygenation of lignin pyrolysis vapors [74], showing that support and oxide state influence which oxygen-removal pathways dominate. Complex copper–acid catalysts can boost conversion of lignin-derived phenolics to cycloalkanes [75], while metal–organic-framework-derived copper materials have been used for lignin hydrogenolysis to monomeric phenols [76]. These examples span different feeds and phases, yet the mechanistic design variable is similar: a surface must activate an oxygenated aromatic, supply reactive hydrogen, and control residence time so that deoxygenation does not automatically become over-hydrogenation. Measuring only final yield hides the sequence. Intermediate-resolved time courses, isotope tracing, and site-specific poisons can expose whether C–O cleavage precedes ring hydrogenation and whether the two events occur on the same ensemble. Because phenolic feeds can strongly adsorb, apparent deactivation may also shift selectivity before conversion changes. Time-resolved product distributions should therefore be paired with coke or oxygen-coverage measurements so that changes in pathway branching can be separated from simple loss of total accessible surface.

### 5.3. Defect-Rich and Nanoreactor Interfaces

Defect-rich metal–oxide perimeters provide another route to cooperation. Ni nanoparticles positioned near oxygen vacancies on CeO_2_ have been used to promote hydrogenolysis of lignin model compounds [77]. Metal–acid interfaces encapsulated within MOF-derived environments have likewise been engineered for hydrogenolysis of biomass-derived aromatic aldehydes [78]. In both architectures, a reducible or acidic support is more than a dispersant: it activates oxygen-containing groups, alters electron density at the metal, and may participate in hydrogen migration. Independent work on phenol hydrogenation over Pd/reducible oxides has directly connected hydrogen spillover and substrate-support hydrogen bonding to reactivity [79]. Although phenol is a simplified model, this mechanism may also be relevant to lignin-derived phenolics, where support hydroxyls and vacancies can influence adsorption geometry and hydrogen availability. Interface density, not simply metal surface area, is therefore a plausible scaling variable for activity and selectivity. Defect concentration should be quantified before and under reaction where possible, because vacancies can heal in water or oxygenated feeds and regenerate under hydrogen. If rate follows this reversible defect population rather than the ex situ vacancy density, the relevant synthesis target becomes the regenerability of defects at the metal–support perimeter.

The spatial dimension of cooperation can extend to mesoscale nanoreactors and external energy fields. Magnetically induced catalytic reduction of biomass-derived oxygenates in water demonstrates that local heating or field-responsive catalyst structures can alter where energy is deposited [80]. Metal@hollow-carbon nanoreactors provide a strategy for positioning metal sites inside confined shells that regulate diffusion and local chemical potential during hydrodeoxygenation [81]. Such architectures can improve selectivity, but they also complicate interpretation because transport, temperature gradients, and chemistry become inseparable. The correct design comparison is not simply conversion per gram of metal; it should include interface-normalized rate, residence-time distribution, internal temperature or concentration gradients where relevant, and accessibility after repeated cycles. A cooperative catalyst is mechanistically credible when its architecture produces a reproducible kinetic advantage that cannot be explained solely by greater surface area or altered transport. For field-assisted or confined reactors, local hot spots can mimic lower apparent activation barriers or altered selectivity. Calibrated thermal mapping and control experiments with equivalent bulk temperature profiles are needed before assigning a chemical effect to magnetic induction, confinement, or localized energy delivery.

## 6. Operando-to-Model Workflows for Resolving Dynamic States

### 6.1. Observing the Working State

**Figure 5:**
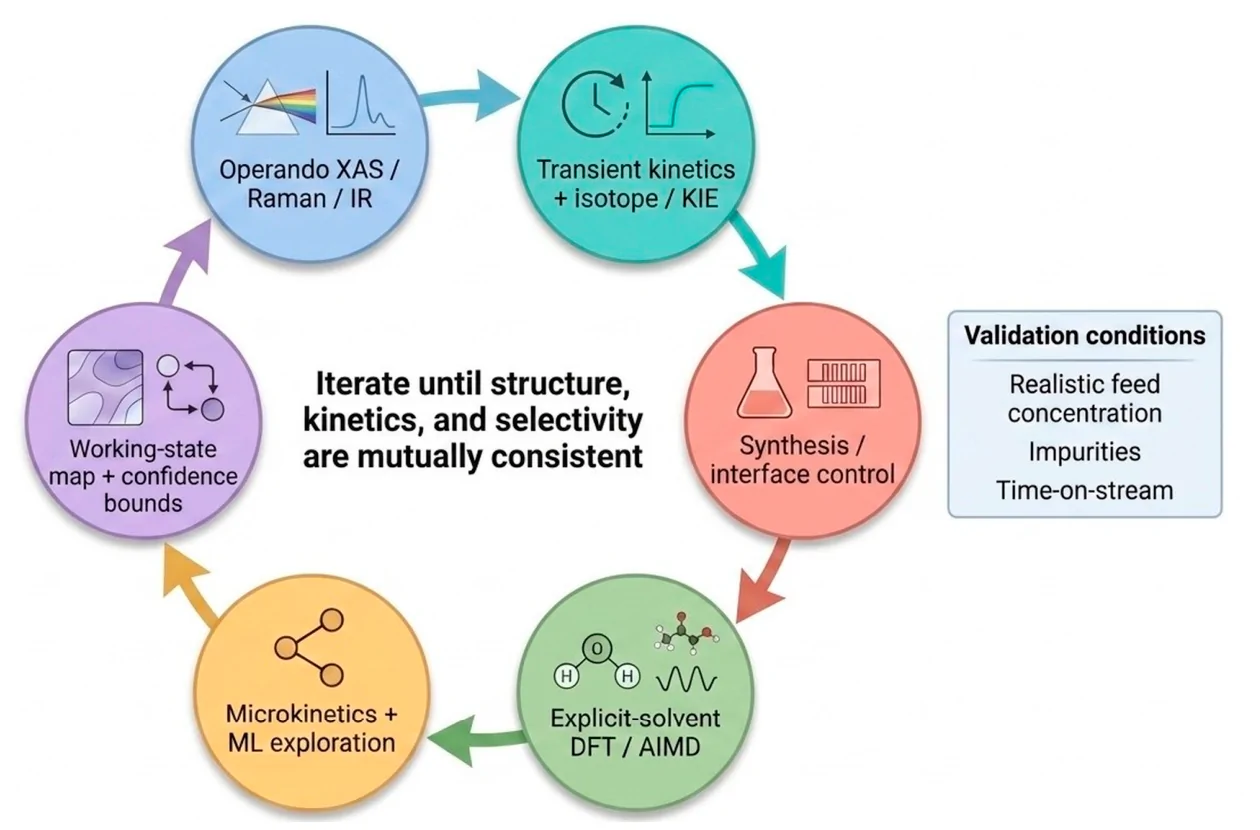
Closed-loop operando-to-model workflow. Operando spectroscopy, transient/isotopic kinetics, explicit-solvent calculations, microkinetic or machine-learning-assisted exploration, working-state mapping, and synthesis/interface control are iteratively coupled to test and refine state-function relationships under realistic conditions.

Operando analysis is the experimental foundation of adaptive-interface design because it observes the catalyst while the reaction establishes its working state. For biomass electrocatalysis, vibrational spectroscopy can identify adsorbed intermediates, changes in surface oxide/hydroxide, and potential-dependent bonding motifs while current and products are measured [5]. Operando X-ray absorption adds element-specific oxidation-state and coordination information, as illustrated by direct monitoring of phase transitions in amorphous nickel oxyhydroxide during HMF oxidation [28]. Neither technique alone fully identifies an active site: vibrational spectra can be dominated by strongly adsorbed spectators, while X-ray absorption averages overactive and inactive atoms. The strongest inference comes when a structural or spectroscopic feature changes with the same perturbation that changes rate or selectivity. Figure 5 therefore places spectroscopy inside a closed loop with kinetics, isotopes, computation, and synthesis rather than treating characterization as a post hoc confirmation step. Spectral acquisition must also be synchronized with product sampling, since a structurally resolved state that appears during a rapid potential sweep may never dominate during preparative electrolysis. Matching the temporal resolution of spectroscopy, electrochemistry, and analytics is therefore part of the mechanistic design rather than a purely instrumental choice.

Time resolution is central because many catalyst transformations occur on the same scale as turnover. A steady-state spectrum can average multiple states and obscure the sequence connecting them. Modulated-excitation and multi-technique operando approaches provide one strategy for extracting the periodically responding component from a large static background [82]. In electrochemistry, potential steps can be combined with rapid spectroscopy and product analysis to determine whether formation of a high-valent state precedes organic oxidation or merely correlates with it. In thermocatalysis, pressure or feed-composition steps can perturb hydrogen coverage and interfacial hydration. The essential output is a response time: if the structural signal changes faster than the rate, it may be a prerequisite state; if it lags product formation, it may be a consequence or deactivation marker. Reporting these timescales prevents the common mistake of assigning activity to the most intense operando species without demonstrating kinetic relevance. Frequency-dependent modulation offers an additional discriminator: species that track fast forcing are likely close to the catalytic cycle, whereas slowly accumulating signals can indicate spectator pools or degradation. This logic is especially useful in concentrated biomass feeds where strong background absorption complicates direct assignment.

### 6.2. Kinetic and Computational Constraints

Transient and isotopic kinetics constrain species that spectroscopy cannot distinguish unambiguously. H/D exchange can test whether surface hydrogen is mobile and whether proton transfer contributes to the rate-limiting sequence. Kinetic isotope effects can distinguish C–H/O–H cleavage involvement, although solvent reorganization and equilibrium isotope effects must be considered in aqueous systems. Reaction orders in H_2_, substrate, water, or base can reveal saturation or inhibition, but orders should be measured within a regime where the working-state population does not change discontinuously. For cooperative interfaces, selective poisoning or spatial dilution can test whether two functions must be adjacent. The water-mediated H_2_ heterolysis proposed for Pd-S interfaces [66] is an example where isotope labeling and water-activity perturbation are mechanistically informative. Similarly, solvent-modulated hydrogen species in furfural hydrogenation [42] can be interrogated by changing donor and solvent isotopic composition while monitoring product-specific rates. For liquid-phase systems, isotope experiments should additionally report exchange with solvent and functional groups before reaction, because rapid pre-equilibration can erase the labeling pattern expected from the catalytic event. Product-specific isotopologue analysis is substantially more informative than a single global isotope effect when parallel pathways coexist.

Explicit-solvent computation is necessary when hydrogen-bond networks and interfacial water reorganize along the reaction coordinate. Atomistic studies of water–solid interfaces show that a small number of static water molecules can be insufficient because the relevant configuration is an ensemble [12]. For furfural, simulations at metal–water interfaces demonstrate that solvation can change adsorption and reaction energetics [43]. A practical hierarchy is to begin with periodic DFT and several explicit solvent configurations, then use ab initio molecular dynamics or enhanced sampling when solvent rearrangement is slow or strongly coupled to bond making/breaking. Free-energy profiles should be compared with experimentally measured temperature dependence or isotope effects where possible. Computational models should also test multiple working-state structures rather than optimize only the ex situ surface. If two states are close in free energy, microkinetics may predict that both contribute under turnover even though only one is thermodynamically dominant in vacuum.

Microkinetic modeling converts elementary-step energetics into steady-state coverages, rates, and selectivity, making it well suited to dynamic interfaces when state interconversion is included explicitly [6,7]. The model should contain reactions that create and consume the working state—oxidation/reduction of a metal center, hydroxylation, vacancy filling, hydrogen spillover, or protonation—in addition to substrate conversion. This prevents a common inconsistency in which DFT barriers are calculated on an assumed active surface whose population is never predicted. Sensitivity analysis can then identify whether rate depends more strongly on substrate activation or on formation of the active state. For electrochemical systems, potential and local pH dependencies must be included; for thermocatalytic systems, solvent chemical potential and H_2_ fugacity are key. Mechanistic models become especially powerful when they predict a non-monotonic optimum in state population that can be tested experimentally by potential, pressure, or composition sweeps. Parameter uncertainty should be propagated to predicted coverages and selectivities rather than hidden by a single best-fit parameter set. When several mechanisms fit steady-state rates equally well, transient response or state-resolved spectroscopy should be selected specifically to discriminate the competing models.

### 6.3. State-Space Exploration and Closed-Loop Design

Data-driven exploration can broaden the state space beyond human-selected pathways. Machine-learning-accelerated automatic process exploration has shown, in a non-biomass Pd oxidation example, that thousands of elementary restructuring events may become accessible on catalytic timescales [83]. The relevance to biomass catalysis is methodological: oxygen-rich adsorbates, water, and hydrogen can create an even larger combinatorial space of surface states than simple gas-phase reactants. A useful workflow is to train interatomic potentials or surrogate models on carefully chosen DFT data, explore candidate restructuring and reaction events, then return the highest-sensitivity states to higher-level calculations and targeted experiments. Such models should not be used as black-box predictors of selectivity without uncertainty estimates. Their value is in discovering plausible states and transitions that conventional static intuition would omit. The final mechanistic assignment must still be constrained by operando and kinetic evidence. The training set should deliberately include hydroxylated, reduced, oxidized, adsorbate-covered, and defect-containing configurations expected under biomass conditions. Otherwise, an apparently accurate potential may interpolate only around an ex situ structure and fail exactly where adaptive restructuring becomes chemically important.

The closed-loop workflow ends by back-propagating the resolved working state into synthesis variables. If operando data show that activity tracks a specific NiOOH population [28], synthesis should maximize rapid, reversible access to that state rather than simply maximize nickel loading. If solvent studies show that a hydrogen-bonded adsorption geometry is selective [44], surface functionalization or pore chemistry should reproduce that local environment. If spillover is limiting [65], interface distance and hydrogen-transfer bridges become synthesis targets. This logic turns characterization into an engineering specification. The loop should be repeated under realistic concentration, impurity, and time-on-stream conditions because the working-state map can shift as the process is intensified. An adaptive catalyst is not proven by one successful operando experiment; it is proven when the same state-function relationship predicts performance across controlled perturbations and remains valid when the feed approaches industrial complexity. For each synthesis iteration, the most useful performance target is a paired metric: the fraction or lifetime of the proposed productive state and the product-specific turnover associated with it. Improvement in only one metric can expose compensation, such as generating more active state while simultaneously lowering its intrinsic selectivity. Figure 5 summarizes this evidence-to-synthesis feedback loop.

## 7. Comparative Evidence Matrix and Design Benchmarks

### 7.1. Evidence-Linked Benchmarking

A comparative evidence matrix is useful because the same apparent performance improvement can arise from different physical causes. Higher HMF oxidation current may reflect more active oxyhydroxide, greater electrochemically accessible area, faster interfacial proton transfer, or simply improved mass transport. Higher furfural selectivity may arise from altered adsorption geometry, changed hydrogen activity, faster product desorption, or suppression of ring adsorption. Table 1 therefore classifies representative working-state phenomena by the evidence used to identify them and by the design lever they imply. The purpose is not to rank catalysts across incompatible reaction conditions, but to separate variables that are often collapsed into one activity metric. A mechanistic benchmark is strongest when a state variable is measured directly and then perturbed independently of total catalyst loading. The matrix also prevents mechanistic vocabulary from becoming a substitute for evidence: terms such as reconstruction, spillover, confinement, and bifunctionality are treated as hypotheses that require an observable, a perturbation, and a kinetic consequence. This makes cross-study comparison possible even when absolute rates were measured under different conditions.

Microenvironment effects require a parallel evidence matrix because bulk solvent descriptors do not identify the interfacial origin of a rate change. Table 2 compares representative microenvironmental and cooperative-interface levers, including solvent-controlled reactive-hydrogen speciation, explicit metal–water solvation, surface hydrogen-bond modifiers, modular H_2_/substrate activation, spillover efficiency, and impurity-sensitive interfaces. Each lever has a characteristic failure mode. For example, increasing hydrophobicity can raise organic access but exclude the water needed for proton shuttling; stronger confinement can increase local concentration but slow product escape; and higher base concentration can accelerate deprotonation while shifting oxyhydroxide state populations. The appropriate optimization variable is therefore mechanistically conditional. Comparing a single conversion value across solvents or supports without measuring adsorption, site accessibility, or local state can generate false structure–activity relationships. A particularly useful diagnostic is to perturb one interfacial variable while holding bulk composition as constant as possible. Surface functionalization at matched porosity, controlled water activity at matched substrate activity, or site-separation series at matched loading can isolate causality more effectively than changing several catalyst and solvent properties simultaneously.

Table 3 organizes the measurement and modeling toolbox around observables rather than instrument prestige. X-ray absorption is strongest for element-specific coordination and oxidation state; vibrational spectroscopy is strongest for adsorbates and local bonding; transient kinetics and isotopes connect state changes to rate; explicit-solvent computation resolves molecular-scale solvation; and microkinetic/data-driven approaches test whether the proposed state distribution can reproduce observed selectivity. No single method is sufficient. A high-quality adaptive-interface study should pair at least one working-state observable with at least one kinetic constraint and, where mechanism depends on solvent or site proximity, one structural or computational measure of the local environment. This triangulation also protects against artifacts introduced by beam exposure, electrochemical cell geometry, catalyst concentration, or simplified model surfaces. The pairing should be chosen according to the suspected ambiguity. If oxidation state is uncertain, combine XAS with potential-step kinetics; if adsorbate geometry is uncertain, combine vibrational/isotope probes with explicit-solvent calculations; if spatial cooperation is uncertain, combine poisoning or dilution with microscopy and transport controls.

### 7.2. Reproducibility and Mechanistic Normalization

The benchmarks also clarify what should be reported for reproducibility. For electrocatalytic biomass conversion, potential should be referenced and corrected consistently, electrolyte composition and substrate concentration should be explicit, and product balances should distinguish Faradaic efficiency from carbon selectivity. For thermocatalytic hydrogenation, H_2_ pressure, stirring or transport checks, catalyst pre-treatment, solvent water content, and substrate concentration should accompany conversion and selectivity. For lignocellulosic fractionation, feedstock composition, particle size, solvent-to-biomass ratio, delignification, monomer yield basis, and carbohydrate retention are needed because catalyst performance is inseparable from fractionation chemistry [1,85]. These reporting elements are not administrative details; they define the chemical potentials that establish the working interface. For lignin-first processing, carbohydrate-pulp quality and downstream usability are increasingly recognized as co-products that constrain acceptable fractionation severity [85]. Reporting only aromatic monomer yield can therefore overstate process performance when the remaining carbohydrate stream has been chemically damaged or becomes difficult to valorize.

A final benchmarking principle is to normalize performance to the descriptor that the mechanism predicts. If the active ensemble is a metal–support perimeter, rate per exposed metal atom may obscure the causal scaling variable. If an oxyhydroxide fraction is active, geometric current normalized by total nickel can mix changes in active-state population with intrinsic kinetics. If confinement controls selectivity, external surface area may be irrelevant. Mechanistic normalization should therefore follow the proposed elementary step and be supported by independent site quantification. Where such quantification is not possible, performance should be reported using multiple normalization approaches, and claims of intrinsic superiority should be avoided. This discipline makes literature comparisons slower but far more transferable for rational design. This is particularly important for reconstructed electrocatalysts because electrochemically active area, redox charge, and productive-state fraction can evolve simultaneously during conditioning. Presenting geometric, mass-normalized, and state-normalized metrics together allows readers to see whether the claimed improvement arises from more sites, different sites, or faster turnover per productive site.

## 8. Mechanism-Guided Design Rules, Scale-Up, and Research Priorities

### 8.1. Working-State and Solvent Co-Design

The first design rule is to synthesize for controlled access to the working state. Defects, strain, ligands, supports, and precursor chemistry should be evaluated by how they change the barrier and thermodynamics of state formation under turnover. Cation-defective nickel hydroxides illustrate the concept because defect engineering can facilitate formation of catalytically active NiOOH while also modifying substrate adsorption [30]. The analogous thermocatalytic strategy is to create interfaces that become selectively hydroxylated, reduced, or vacancy-rich only under reaction conditions. Stability then means repeatable state cycling without irreversible loss of interface density. Screening workflows should therefore include conditioning cycles and reversible perturbations before ranking materials. A catalyst that reaches high activity only after substantial uncontrolled reconstruction may be difficult to manufacture reproducibly even if its steady-state performance is excellent. Precursor libraries should consequently be ranked after identical conditioning histories and with the active-state fraction measured at the same conversion or current. This avoids selecting materials merely because one precursor transforms faster during the initial experiment, while another reaches a more stable and selective steady state after conditioning.

The second rule is to co-design solvent and catalyst rather than optimize them sequentially. Solvent selection changes adsorption, active hydrogen speciation, local proton activity, product stability, and downstream separations [3,42,59]. The best material in one solvent may not be the best in another because the solvent changes the state distribution of both catalyst and substrate. A practical workflow is to identify the elementary step requiring the greatest stabilization or transport assistance, choose a solvent or mixed solvent that creates that local environment, and then design surface functionality to retain it at the interface. Water activity should be treated as a variable even in nominally organic media because trace water can reorganize acid–base chemistry. At process scale, the solvent must also tolerate recycle-derived impurities and integrate with separation or biological upgrading [64]. Solvent screens should include at least one mechanistic observable in addition to yield, such as adsorption strength, hydrogen/deuterium exchange, interfacial spectroscopy, or measured wettability. That extra variable can distinguish a solvent that truly stabilizes a desired transition state from one that simply changes substrate availability or product extraction.

### 8.2. Cooperative Functions and Realistic Feeds

The third rule is to match rates across cooperative functions. Bifunctional catalysts should be designed around fluxes of intermediates rather than counts of nominal site types. If hydrogen dissociation is much faster than substrate activation, excess hydrogen coverage can promote secondary reactions; if substrate activation is fast but hydrogen delivery is slow, strongly bound intermediates can foul the surface. Modular furfural hydrogenation demonstrates the advantage of assigning H_2_ activation and substrate activation to complementary functions [46], while spillover engineering shows that transport between those functions can itself be rate-limiting [65]. Synthesis should therefore vary site ratio and separation independently whenever possible. Spatially resolved characterization, selective poisoning, and dilution experiments can identify the distance over which cooperation remains effective. The optimum is a kinetic match, not necessarily a 1:1 compositional ratio. When this distance dependence is known, synthesis can move from empirical component mixing to deliberate interface-density control. The relevant scale may be atomic for heterolytic H_2_ activation, nanometric for spillover, or mesoscopic when a soluble intermediate shuttles between domains, and the required characterization should match that scale.

The fourth rule is to design for feed complexity before finalizing catalyst composition. Authentic biomass streams contain water, salts, acids, oligomers, sulfur- or nitrogen-containing species, and upstream solvent residues that can change adsorption or state evolution. Trace-impurity effects in HMF hydroconversion demonstrate that seemingly minor components can strongly perturb catalytic behavior [62]. Biomass-derived electrolytes similarly alter furfural electrohydrogenation relative to idealized media [41]. A staged validation protocol should therefore progress from purified model molecule to controlled impurity mixtures, concentrated feeds, and finally process-derived streams. Operando or rapid ex situ state diagnostics should be repeated at each stage. A catalyst that maintains conversion while switching to a less selective working state is not robust; a catalyst that preserves the same state-selectivity relationship across feed complexity has stronger scale-up credibility. A useful robustness map plots selectivity against impurity identity and concentration while simultaneously tracking a state-sensitive signal. Such maps can reveal threshold behavior, identify reversible poisons, and prioritize feed-cleanup operations only for impurities that actually push the catalyst out of its productive working-state window.

### 8.3. Reactor Integration, Lifetime, and Uncertainty

The fifth rule is to couple catalyst architecture to reactor transport. Structured catalysts and additive manufacturing can control channel geometry, heat transfer, pressure drop, and spatial placement of catalytic functions [13]. Membrane hydrogenation shows that hydrogen delivery can be decoupled from bulk-liquid saturation [54], and hollow nanoreactors can tune residence time and local concentration [81]. These architectures are valuable only when transport is quantified sufficiently to separate physical intensification from intrinsic chemistry. Dimensionless analysis, stirring tests, particle-size variation, permeability measurements, or spatial temperature sensing should accompany claims of interface-driven improvement. At larger scale, catalyst state can vary along a reactor because substrate, hydrogen, water activity, and temperature vary axially. Reactor design should therefore seek a controlled working-state profile, not merely uniform catalyst composition. Scale-up experiments should also test whether the same state-function relation survives gradients in substrate conversion along the reactor. Spatially resolved sampling or staged catalyst beds can identify where the interface changes state and whether redistributing catalytic functions can maintain the preferred state over a larger fraction of the reactor volume.

The sixth rule is to make deactivation mechanistic and reversible where possible. Coffee waste-biochar-supported copper electrocatalysis has recently been used to probe surface dynamics and catalyst restructuring during biomass depolymerization [84], illustrating that waste-derived supports can themselves participate in state evolution. For conventional catalysts, irreversible sintering, leaching, coking, and pore blockage should be separated from reversible redox or hydration shifts by regeneration experiments. If activity returns after restoring potential, hydrogen pressure, or solvent composition, the apparent deactivation may be a state transition rather than material loss. If it does not, microscopy, elemental analysis, and surface spectroscopy should quantify the lost ensemble. Lifetime models should then track the descriptor tied to mechanism—interface length, vacancy density, active oxyhydroxide fraction, or accessible acid–metal pairs—instead of total catalyst mass. Regeneration protocols should be designed around the identified failure mode: oxidative removal for carbonaceous deposits, controlled reduction for over-oxidized sites, solvent washing for strongly retained organics, or re-hydroxylation when loss of interfacial water is implicated. Blind regeneration can restore conversion while creating a different selectivity profile.

The seventh rule is to use uncertainty as a design variable. Dynamic-interface models contain uncertainty in structure, coverage, solvation, and kinetic parameters. Rather than selecting one favored pathway, computational and experimental programs should identify which uncertain parameter most strongly affects predicted selectivity, then design the next measurement to reduce that uncertainty. Multi-technique operando strategies [82] and machine-learning-assisted process exploration [83] are complementary in this respect: one constrains the state space experimentally, while the other searches states and transitions that conventional intuition may miss. The resulting workflow is an active-learning loop for catalysis. Its output should be a state map with confidence bounds and experimentally testable transitions, not a single cartoon mechanism. Such maps are better suited to scale-up because they reveal where changes in concentration, solvent activity, or temperature may push the catalyst across a state boundary. Reporting uncertainty also discourages false precision in computed barriers and fitted kinetic constants. More importantly, it identifies the experimental region where a small change in solvent activity, potential, or defect population is predicted to cause a large selectivity change, which is exactly where targeted validation has the highest information value.

## 9. Conclusions

Biomass valorization increasingly operates in regimes where the catalyst, solvent, adsorbates, and driving force form a coupled reactive interface. The literature reviewed here shows that phase transitions in nickel oxyhydroxides, solvent-dependent active hydrogen, water-mediated hydrogen heterolysis, support hydrogen bonding, defect-assisted adsorption, and hydrogen spillover can each change selectivity without altering the nominal catalyst label. These are not isolated exceptions; they are manifestations of the same principle that the working state is conditioned by turnover. A catalyst should therefore be described by the states it occupies under defined chemical potentials and by the rates at which it moves among them. This representation is more mechanistically useful than an ex situ composition alone and provides a common language across thermocatalytic and electrocatalytic biomass upgrading. This perspective also explains why apparently contradictory literature trends can both be valid: different studies may prepare nominally similar catalysts but establish different working states because pre-treatment, solvent, concentration, potential, or impurity history differs. Reproducible catalysis therefore requires reproducible state preparation, not only reproducible synthesis.

The most transferable design strategy is a closed loop connecting synthesis, operando observation, kinetics, atomistic modeling, and state-resolved benchmarking. Operando spectroscopy identifies candidate working states; transient and isotope experiments test kinetic relevance; explicit-solvent models determine how the liquid environment reshapes adsorption and transition states; and microkinetic or data-driven exploration tests whether the proposed state network can reproduce measured behavior. The inferred state-function relation then becomes a synthesis specification: stabilize a particular interface, tune defect formation, control wettability, shorten spillover distance, or engineer a proton relay. Repeating the loop under concentrated and impurity-containing feeds converts mechanistic insight into process robustness. The loop should be considered successful only when it predicts the direction of a new perturbation that was not used to construct the mechanism. Prospective prediction—for example, how selectivity changes with water activity or controlled site separation—is a stronger test of rational design than retrospective fitting of an existing catalyst series.

The central opportunity is to move from catalyst discovery toward catalyst-state engineering. For HMF, furfural, lignin-derived oxygenates, and lignocellulosic fractionation, future advances are likely to come from controlling when and where reactive states appear, how solvent and confined water organize around them, and how complementary sites exchange hydrogen, protons, electrons, or adsorbed intermediates. This approach does not discard conventional descriptors such as particle size, acidity, or composition; it embeds them in a causal framework that explains why the same material can behave differently across solvents, potentials, feed concentrations, and time-on-stream. Adaptive catalytic interfaces therefore offer a practical route to more selective, durable, and scalable biomass conversion while imposing a higher standard of mechanistic evidence for claims of rational design. For the field, this shift favors studies that report dynamic descriptors alongside conventional composition and that preserve negative results when a proposed state-function relation fails. Such evidence will make adaptive-interface principles transferable across feedstocks and will reduce the number of catalyst formulations that appear unique only because they were tested under incomparable conditions.

## Figures and Tables

**Table 1:** Representative dynamic working states and evidence-linked design implications.

Working-State Phenomenon	Representative Reaction/System	Evidence Linked to Function	Design Implication
NiOOH ↔ Ni(OH)_2_ phase cycling	HMF electrooxidation	Operando XAS/Raman resolves reaction-coupled phase transition and kinetics [28]	Tune precursor/defects for rapid reversible access to productive oxyhydroxide.
Cation-defect-assisted active-state formation	HMF electrooxidation	Defects promote Ni(OH)_2_-to-NiOOH conversion and HMF adsorption [30]	Engineer defect density together with substrate-binding strength.
Surface reconstruction under cathodic bias	HMF electrohydrogenation on Ag	In situ reconstruction correlates with competent hydrogenation activity [35]	Rank catalysts after electrochemical conditioning, not only as-synthesized facets.
Hydration-controlled H_2_ heterolysis	Hydrodehydroxylation at Pd-S	Water-mediated heterolytic activation proposed from interface-specific evidence [66]	Control perimeter chemistry and local water activity.
Dynamic biomass-depolymerization surface	Cu/biochar electrocatalyst	Surface dynamics and restructuring observed during biomass depolymerization [84]	Track support/metal evolution during realistic feed exposure.
Catalytic-timescale metal restructuring	Pd oxidation model system	ML-assisted exploration identifies low-barrier restructuring pathways [83]	Include state interconversion in mechanistic models and uncertainty analysis.

**Table 2:** Microenvironment and cooperative-interface levers for biomass catalysis.

Microenvironment/Cooperative Lever	Mechanistic Effect	Representative Evidence	Failure Mode/Diagnostic
Solvent-controlled active H species	Changes identity/population of reactive hydrogen	Furfural hydrogenation exhibits solvent-modulated multiple active H species [42]	Apparent solvent trend may reflect changed working state; use isotopes and spectroscopy.
Explicit metal–water solvation	Changes adsorption geometry and free energy	Furfural at metal–water interfaces shows solvation-dependent energetics [43]	Continuum solvent may miss H-bond rearrangement; sample multiple solvent configurations.
Surface H-bond modifiers	Biases substrate orientation/transition-state stabilization	Organic modifiers alter furfuryl-alcohol ring hydrogenation through H-bonding [44]	Modifier can block sites; separate site-density loss from intrinsic selectivity.
Modular H_2_/substrate activation	Separates hydrogen generation from carbonyl activation	Furfural hydrogenation improved by modularized functions [46]	Mismatched rates or excessive separation suppress cooperation.
Spillover bridge efficiency	Controls H transfer between source and sink sites	Organic decoration enhances H spillover and hydrogenation [65]	Prove migration with distance/isotope/poisoning tests, not conversion alone.
Impurity-sensitive interface	Competitive adsorption or state poisoning	Trace impurities strongly affect HMF hydroconversion [62]	Validate with controlled impurity additions and authentic feed.

**Table 3:** Operando, kinetic, and modeling toolbox for adaptive-interface studies.

Tool	Primary Observable	Strength for Adaptive Interfaces	Limitation/Best Pairing
Operando XAS	Element-specific valence and local coordination	Tracks active-state formation under turnover; phase-fraction trends [28]	Bulk-averaged signal; pair with surface-sensitive spectroscopy and kinetics.
Operando Raman/IR	Adsorbates, bonds, surface oxyhydroxide signatures	Links molecular intermediates to potential or feed perturbation [5]	Strong spectators may dominate; pair with isotope labeling/product rates.
Transient/isotopic kinetics	Response times, KIE, exchange, site coupling	Tests whether a candidate state participates in rate-determining chemistry	Requires state stability during perturbation; pair with time-resolved spectroscopy.
Explicit-solvent DFT/AIMD	Adsorption/free-energy landscape with water/H-bonding	Resolves solvent participation missing from vacuum models [12,43]	Sampling cost; validate against isotope/temperature/solvent trends.
Microkinetic modeling	State populations, coverages, sensitivity, selectivity	Tests consistency of elementary-step network with steady-state data	Only as complete as state network; include active-state creation/decay explicitly.
ML-assisted process exploration	Large restructuring/event space	Discovers low-barrier state transitions outside hand-selected mechanisms [83]	Requires uncertainty control and experimental validation.

## Data Availability

No new data were created or analyzed in this study. Data sharing is not applicable to this article.
